# Characterization and Hypoglycemic Activity of a Rhamnan-Type Sulfated Polysaccharide Derivative

**DOI:** 10.3390/md17010021

**Published:** 2019-01-01

**Authors:** Jie-Fen Cui, Han Ye, Yu-Jie Zhu, Yin-Ping Li, Jing-Feng Wang, Peng Wang

**Affiliations:** 1 College of Food Science and Engineering, Ocean University of China, Qingdao 266003, China; cjfouc@163.com (J.-F.C.); 17852720293@163.com (H.Y.); zyjbmgf@163.com (Y.-J.Z.); fmsboc@163.com (Y.-P.L.); jfwang@ouc.edu.cn (J.-F.W.); 2 College of Marine Science and Biological Engineering, Qingdao University of Science and Technology, Qingdao 266042, China

**Keywords:** sulfated polysaccharide derivative, structure characterization, safety evaluation, type 2 diabetes, molecular mechanism

## Abstract

Polysaccharide chromium (III) derivatives are gaining increasing attention in improving type 2 diabetes. In this study, the sulfated polysaccharide from *Enteromorpha prolifera* (SPE) with 4.8 kDa was prepared by specific enzymatic hydrolysis. The obtained SPE was used to prepare a rhamnan-type sulfated polysaccharide derivative (SPED). Results indicated that O-H, C=O, and S=O were effectively involved in the chelation of SPED (chromium content 20.26%). Acute (half lethal dose > 2.38 g/kg) and sub-acute toxicity showed that SPED had no damaging effects on mice. Anti-diabetic experiment demonstrated that SPED improved glucose metabolism. Moreover, SPED promoted the PI3K/PKB/GSK-3β signaling pathway by regulating mRNA expression of insulin receptors (IR), insulin receptor substrate 2 (IRS-2), phosphatidylinositol 3 kinase (PI3K), protein kinase B (PKB), and glycogen synthase kinase 3β (GSK-3β). In conclusion, the SPED might represent a novel marine-derived candidate against hyperglycemia, which may undergo further pharmaceutical development as a hypoglycemic agent.

## 1. Introduction

Type 2 diabetes is characterized by chronic metabolic dysregulation of glucose and lipid metabolism, which is closely related to renal failure, cardiovascular disease, and blindness [1]. The International Diabetes Federation reports that the population with diabetes mellitus will reach up to 366 million by 2030 [2]. The increasing prevalence of type 2 diabetes has driven the development of pharmaceuticals and functional foods for treatment. The main therapeutic goal for type 2 diabetes is to achieve repair of impaired pancreatic islets that would consequently ameliorate hyperglycemia [3].

Chromium is an essential mineral in the human body, and chromium (III) is considered the necessary active form for normal glucose and lipid homeostasis in organisms [4]. The potential mechanism of chromium (III) regulation of glucose metabolism is through the intracellular insulin signaling pathway via the activation of phosphorylation of protein kinase B [5]. The low absorption rate of chromium (III) salts has pushed for the synthesis of organic chromium complexes. At present, chromium picolinate is more bioavailable than other commercially organic chromium (III) supplements. However, it was reported that chromium picolinate may induce oxidative stress and genotoxicity [6]. Therefore, developing safe chromium (III) supplements is crucial for improving type 2 diabetes.

*Enteromorpha prolifera*, one of the most common green algae, is distributed along the coastal zone. Owing to its edibility and medicinal properties, *E. prolifera* has been widely used in food and medical fields [7]. Polysaccharides from *E. prolifera* possess significant bioactivities such as antitumor, hypolipidemic, and anticoagulant properties [8]. It has been reported that polysaccharides from *E. prolifera* are excellent metal-ion chelating ligands, and its oligosaccharides usually possess appreciable aqueous solubility and bioactivity [9].

In this study, sulfated polysaccharide from *E. prolifera* (SPE) with low molecular weight was prepared by a specific degradase. The obtained SPE was used to prepare a rhamnan-type sulfated polysaccharide derivative (SPED). The physicochemical properties and the effects of SPED on hyperglycemia were investigated in mice. This study would provide experimental evidence on SPED potential for ameliorating type 2 diabetes. 

## 2. Results and Discussion

### 2.1. Characterization of SPE 

**Figure 1:**
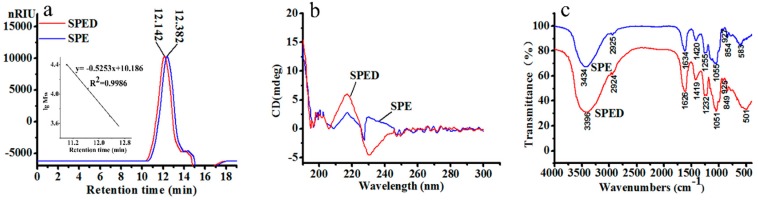
HPGPC chromatogram (**a**), CD spectrum (**b**), and FTIR spectrum (**c**) of SPE and SPED (color artwork).

As shown in Table 1, the sulfate ester content of SPE was 25.9%, respectively. The main monosaccharide of SPE was rhamnose (59.8%). The SPE with 4.8 kDa possessed good aqueous solubility, while the viscosity of sulfated polysaccharide was very high. Zhang et al. (2010) reported that polysaccharides from persimmon with high molecular weight and viscosity were difficult to pass through organizational barriers, which limited their biological activities [10]. Combo et al. (2013) have reported that oligosaccharides from sugar beet possess appreciable aqueous solubility and bioactivity [11]. Shi et al. (2013) prepared four polysaccharides with different molecular weights (151.7, 64.5, 58.0, and 28.2 kDa) from *Ulva pertusa*, and the polysaccharide-iron (III) complex with low molecular weight was more effective in protecting mice from radiation induced damage of bone marrow cells [12]. In this study, SPE appeared as a single and symmetrical peak in high-performance gel permeation chromatography (HPGPC) (Figure 1a), which indicated its high homogeneity. Based on the results analyzed above, SPE was chosen as the ligand to prepare the SPED.

### 2.2. Characterization of SPED 

The SPED was synthesized under optimum conditions (data not shown). It was a green powder (total sugar 71.6%) with good water solubility. As shown in Table 1, the sulfate ester content of SPED (24.38%) was very similar to that of SPE (25.9%). The SPED also consisted of four monosaccharides, including rhamnose (58.2%), glucose (4.3%), xylose (16.9%), and glucuronic acid (20.5%). As shown in Figure 1a, the molecular weight of the SPED (6.4 kDa) was higher than that of SPE (4.8 kDa). A similar result was also obtained by Wang et al. (2017) [13]. Therefore, the chelation reaction between Cr^3+^ and SPE led to the increase of molecular weight. 

The CD spectrum is an effective method to study the three-dimensional structure of compounds [14]. The SPED appeared to have positive and negative Cotton effects at 217 and 230 nm, respectively (Figure 1b). The positive Cotton effect at 217 nm was related to the enhancement of the structure asymmetry of the polysaccharide [15]. Simultaneously, the negative Cotton effect of the new complex appearing at 230 nm might be attributed to the charge transfer interaction between metal ions and the carboxyl group in the polysaccharide chain [16]. The FTIR spectra of SPE and the SPED are shown in Figure 1c. The characteristic absorption bands of SPE at 3434 cm^−1^, 1634 cm^−1^, and 1255 cm^−1^ were generated by the stretching vibrations of O-H, C=O, and S=O, respectively [17,18]. After the chelation with Cr^3+^, some absorption bands of SPED were obviously shifted. Specifically, the band of stretching vibration of O-H shifted from 3434 cm^−1^ to 3396 cm^−1^, which indicated the formation of coordination bond between chromium and hydroxyl of the polysaccharide. In addition, the stretching vibration absorption bands of C=O and S=O also moved towards low wavenumbers, which demonstrated that the carboxyl and sulfate groups were involved in the chelation. These results suggested that a novel rhamnan-type sulfated polysaccharide derivative was synthesized successfully.

Additionally, the chromium content (20.26%) of SPED was relatively higher, compared with that of an *Inonotus obliquus* polysaccharide-chromium (III) complex (13.01%) [13]. Chromium content of SPED might be closely related to the unique structure of the carbohydrate chain. Based on our previous research, the backbone of the polysaccharides from *E. prolifera* consisted of d-GlcUAp-α-(1→4)-3-sulfate-l-Rhap-β-(1→4)-d-Xylp-β-(1→4)-3-sulfate-l-Rhap units, with sulphate ester linked on C-3 position of Rha [19]. Consequently, the polyanionic property of sulfated polysaccharide effectively contributed to the chelating reaction between Cr^3+^ and SPE.

### 2.3. Safety Evaluation of SPED

The acute and sub-acute toxicity of SPED were evaluated according to the methods reported by Saheed et al. (2015) [20]. During the acute toxicity experiment, no significant clinical signs of toxicity were observed. The administration of the SPED at all doses did not lead to any mortality, which showed that the half lethal dose of SPED for ICR male mice was higher than 2.38 g/kg. Wu et al. (2011) reported that organic chromium complexes (half lethal dose > 2 g/kg) were evaluated as non-toxic [21]. In addition, the half lethal dose (0.2 g/kg) of inorganic chromium (http://www.atsdr.cdc.gov/toxprofiles/tp7.pdf) is significantly lower, when compared with that of the SPED. In contrast to inorganic chromium salts, the SPED is relatively safe. 

**Figure 2:**
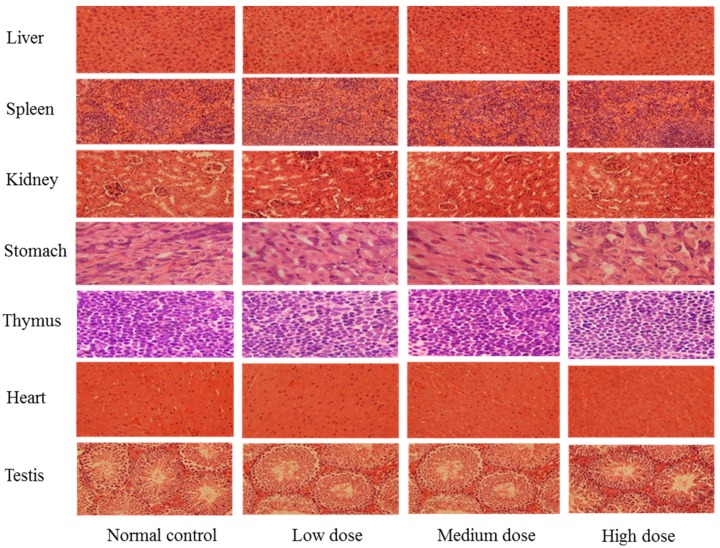
Photomicrographs of organs in normal mice (H&E stain, 40×, color artwork).

To further evaluate the safety of SPED, a sub-acute toxicity experiment was carried out. During the four-week experiment, no dead mice were observed in all groups. The histopathological result confirmed that SPED could not lead to tissue lesions of the liver, spleen, kidney, stomach, thymus, heart, and testis (Figure 2). Results suggested that SPED had no damaging effects on the bodies of normal mice. The coordinated ligand plays a significant role in toxicity evaluation of different kinds of chromium complexes [22]. Chromium complexes with certain ligands might result in some toxicity. As the most widely applied chromium complex, chromium picolinate had been proved to generate hydroxyl radicals and oxidative DNA damage [23,24]. Moreover, the toxicity of chromium picolinate was closely related to the imine ligands of picolinic acid. Alternatively, the ligand of the SPED is a kind of active polysaccharide, so it could be a safe candidate for a chromium supplement.

### 2.4. Anti-Diabetic Effects of SPED

#### 2.4.1. Oral Glucose Tolerance Test

**Figure 3:**
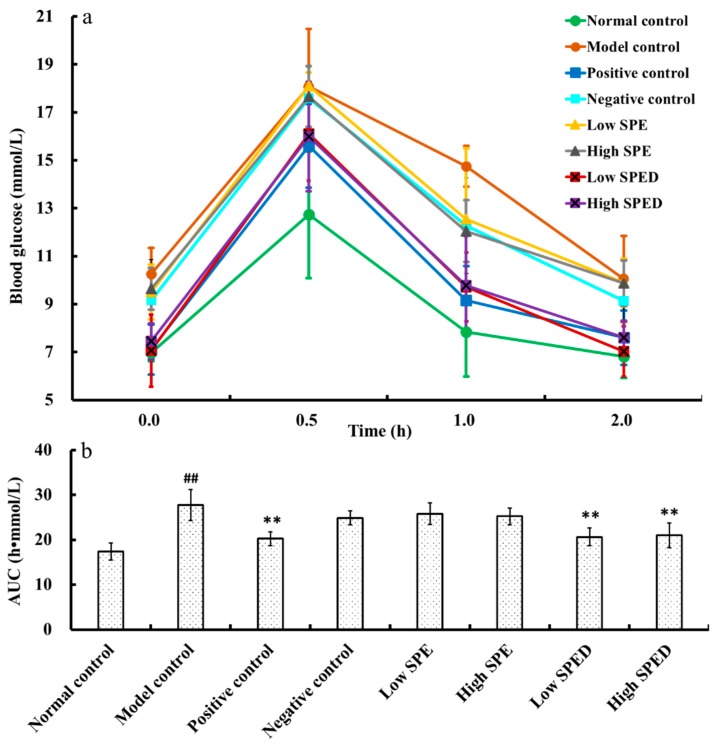
Blood glucose content (**a**), and AUC for oral glucose tolerance (**b**) of the mice (color artwork). ^#^ *p* < 0.05, ^##^ *p* < 0.01 compared with normal control. * *p* < 0.05, ** *p* < 0.01 compared with model control.

The blood glucose content of all groups is shown in Figure 3a. The fasting blood glucose (FBG) content of the model control group (10.75 mmol/L) increased significantly (*p* < 0.01) compared to that of the normal control group (6.97 mmol/L). This result showed that the diabetic model was established successfully. The oral supplementation of glucose led to gradual increase of the blood glucose content within 0.5 h. The blood glucose contents of the positive control, low SPED, and high SPED groups decreased to normal levels after 2 h. In addition, the calculated area under the curve (AUC) value of all groups is shown in Figure 3b. The oral glucose tolerance is negatively related to AUC. The AUC value of the model control group was significantly higher (*p* < 0.01) than that of the normal control group, which indicated that the oral glucose tolerance of the model control group was impaired. It is generally accepted that insulin resistance is closely related to the disorder of glucose metabolism, and long-term hyperglycemia usually triggers microvascular disease [25]. Compared with that of the model control group, AUC values of the two dose groups of the SPED decreased significantly (*p* < 0.01), which suggested that oral glucose tolerance was ameliorated. Wang et al. (2017) reported that *Inonotus obliquus* polysaccharides-chromium (III) complex (900 mg/kg) improved the oral glucose tolerance of type 2 diabetes mellitus mice, using the method of intragastric administration [13]. In the oral glucose tolerance test, the dose-dependent relationship was not observed between the low SPED group and the high SPED group. This suggested that supplementation with SPED (5 mg/kg) could effectively improve the glucose tolerance capacity of diabetic mice. 

#### 2.4.2. Serum Insulin Level

**Figure 4:**
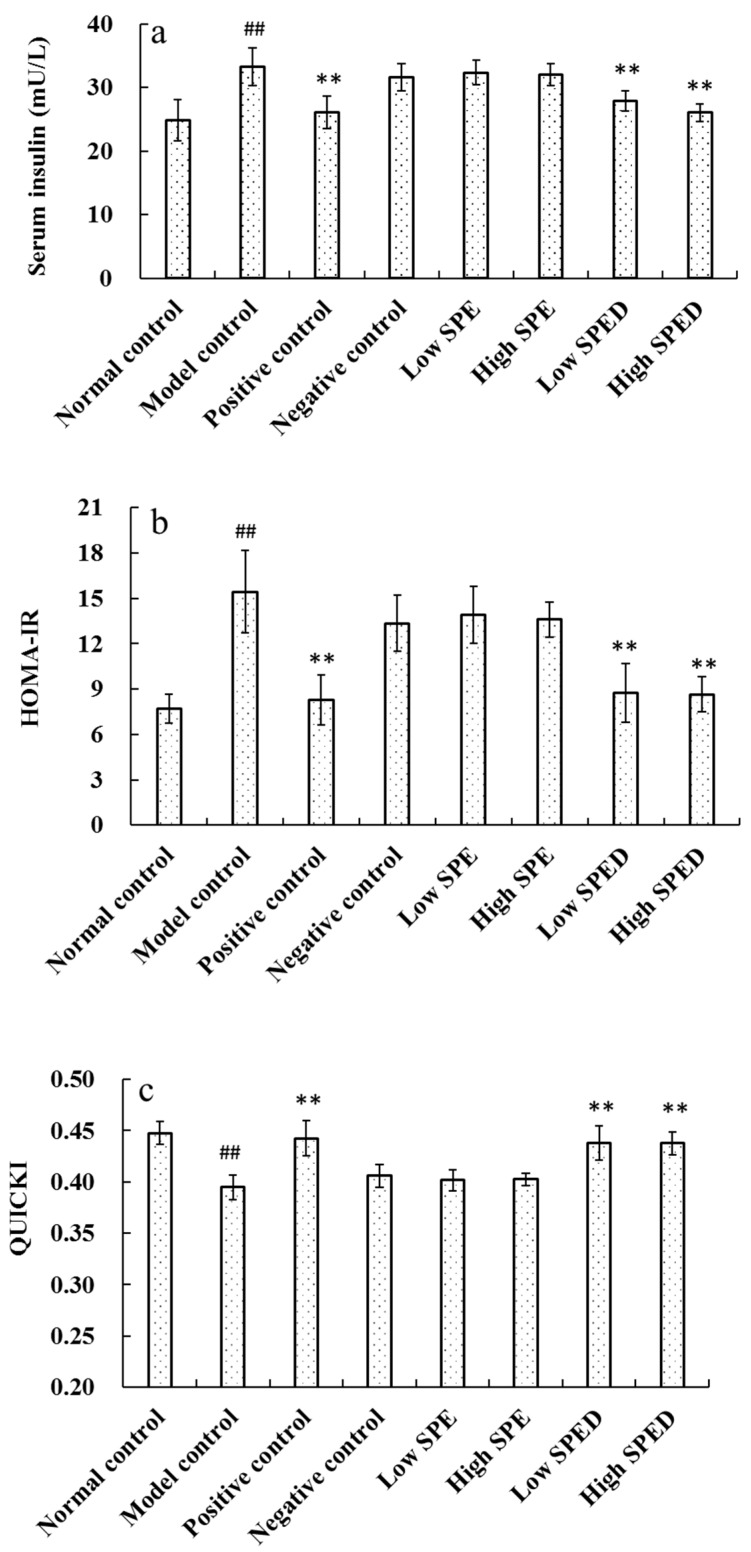
Effects of SPED on the serum insulin (**a**), HOMA-IR (**b**), and QUICKI (**c**) of the mice. ^#^ *p* < 0.05, ^##^ *p* < 0.01 compared with normal control. * *p* < 0.05, ** *p* < 0.01 compared with model control.

As shown in Figure 4a, the serum insulin level of the model control group increased significantly (*p* < 0.01), in comparison with that of the normal control group. Insulin is the primary hormone to regulate glucose metabolism, and it is well known that hyperinsulinism is the major assessment criterion of insulin resistance [26]. In this study, the high-energy diet induced a hormonal disorder in the mice. When mice were given different doses of chromium supplements, the serum insulin level decreased significantly (*p* < 0.01). Apparently, the serum insulin level of the high SPED group approached to that of the positive control. The homeostasis model assessment of insulin resistance index (HOMA-IR) and quantitative insulin sensitivity check index (QUICKI) of all groups are shown in Figure 4b,c, respectively. Compared with that in the normal control group, HOMA-IR in the model control group increased significantly (*p* < 0.01), while QUICKI decreased significantly (*p* < 0.01). These results show that the high-fat and high-sucrose diet induces severe insulin resistance. The HOMA-IR of low SPED group (5 mg/kg) decreased significantly (*p* < 0.01) while QUICKI increased significantly (*p* < 0.01), when compared to that of model control group. Furthermore, QUICKI of low SPED group was similar to that of the positive control group (chromium picolinate). This proved that SPED improved the insulin sensitivity of insulin-resistant mice effectively. Specifically, insulin sensitivity is closely related to the function of pancreatic β-cells [27]. Overall, we assumed that SPED might enhance insulin sensitivity by ameliorating the dysfunction of pancreatic β-cells.

#### 2.4.3. Microscopic Structures of Pancreas Islets

**Figure 5:**
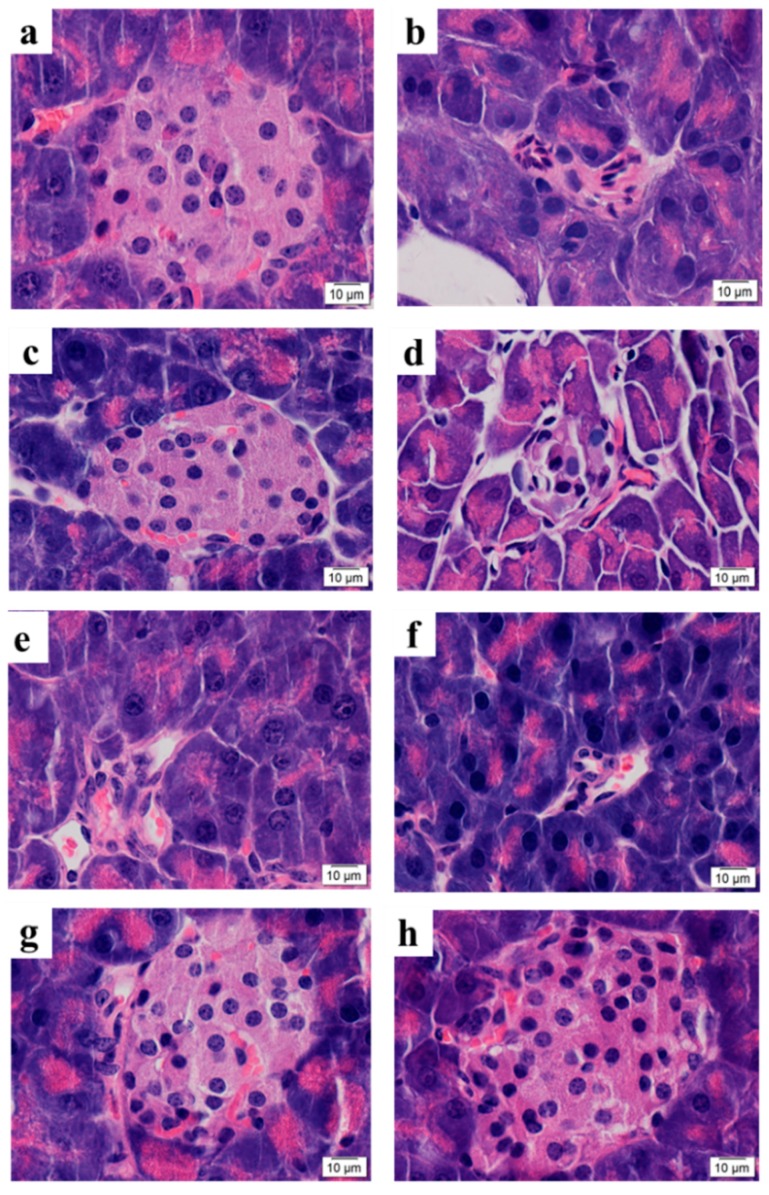
Microstructure of pancreatic islets in mice (H&E stain, 100×) Normal control (**a**), Model control (**b**), Positive control (**c**), Negative control (**d**), Low SPE (**e**), High SPE (**f**), Low SPED (**g**), High SPED (**h**) (color artwork).

To investigate the effects of SPED on pancreatic β-cells, the microstructure of the pancreas islets was photographed (Figure 5). As shown in Figure 5b, multiple pathological changes such as atrophy of the pancreas islets, cell necrosis, and uneven distribution of nuclear chromatin were found in the model control group, compared with the normal control group (Figure 5a). This result showed that insulin resistance induced the dysfunction of pancreatic β-cells. The reduction and necrosis of pancreatic β-cells are the pathological features of type 2 diabetes mellitus [28]. None of the negative control (Figure 5d), low SPE (Figure 5e), and high SPE (Figure 5f) groups produced a significant change in the appearance of the pancreas islets, compared with the model control group. On the contrary, the histological changes of insulin-resistant mice were significantly alleviated, and pancreas islets recovered to a normal rounded appearance, after treatment with SPED (Figure 5g,h). Pancreatic β-cells are specialized cells that produce the insulin required by an organism to maintain glucose homeostasis [29]. Hu et al. (2013) reported that the combination of fucosylated chondroitin sulfate and rosiglitazone improved insulin sensitivity, and no histopathological change of the pancreas islets was observed [30]. In contrast, photomicrographs for the pancreas islets confirmed that SPED restored the impaired pancreatic β-cells and maintained the integrity of pancreas islets. 

#### 2.4.4. Hepatic Glycogen Content

**Figure 6:**
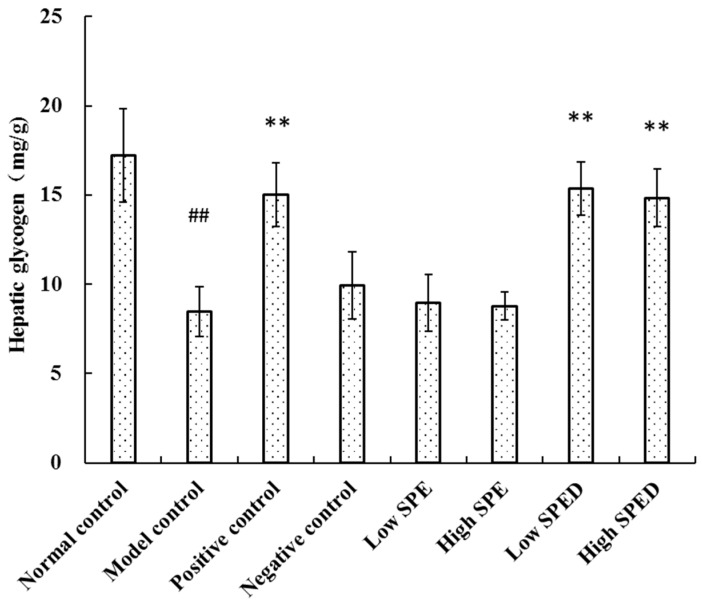
Effects of SPED on hepatic glycogen content of the mice. ^#^ *p* < 0.05, ^##^ *p* < 0.01 compared with normal control. * *p* < 0.05, ** *p* < 0.01 compared with model control.

The liver plays a key role in metabolic homeostasis, and it is a major site for the synthesis, metabolism, storage, and redistribution of carbohydrates. The hepatic glycogen content of the model control group decreased significantly (*p* < 0.01) in comparison with that of the normal control group (Figure 6). The reduction rate in hepatic glycogen of the model control group was 50.73%, which demonstrated that hepatic glycogen synthesis was blocked. The content of hepatic glycogen of the two SPED groups increased significantly (*p* < 0.01) after chromium supplementation. Hepatic glycogen is the primary intracellular storage form of glucose in liver, and its level may be related to the activities of glycogen synthase and glycogen phosphorylase [31]. These results illuminated that the SPED could alleviate hyperglycemia by promoting the synthesis of hepatic glycogen in insulin-resistant mice.

#### 2.4.5. Quantitative Real-Time PCR Analysis

**Figure 7:**
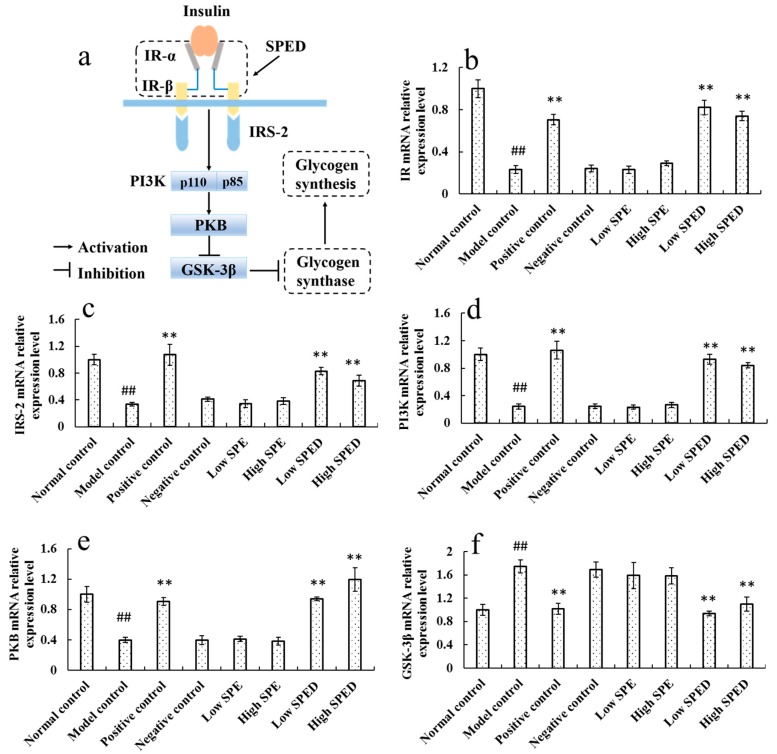
Proposed molecular mechanism of SPED in improving hyperglycemia (**a**), and mRNA relative expression of IR (**b**), IRS-2 (**c**), PI3K (**d**), PKB (**e**), and GSK-3β (**f**) in the PI3K/PKB/GSK-3β pathway (color artwork). ^#^ *p* < 0.05, ^##^ *p* < 0.01 compared with normal control. * *p* < 0.05, ** *p* < 0.01 compared with model control.

Hepatic glycogen synthesis is a crucial metabolic pathway involved in blood glucose homeostasis and metabolism, which is regulated by insulin. It is generally acknowledged that the PI3K/PKB/GSK-3β signaling pathway is of vital importance in the synthesis of hepatic glycogen [32]. In the PI3K/PKB/GSK-3β signaling pathway (Figure 7a), IR is a tetrameric protein, consisting of two extracellular α subunits and two transmembrane β subunits. Insulin binding to α subunits induces a conformational change in the β subunits, which increases catalytic activity of IR [33]. The activated IR then phosphorylates tyrosine residues of IRS-2. With tyrosine phosphorylation, IRS-2 interacts with the p85 regulatory subunit of PI3K, resulting in the activation of PI3K and PKB in sequence. Moreover, PI3K and PKB are the pivotal protein kinases, which regulate the activity of GSK-3β negatively [34]. As shown in Figure 7b, the IR mRNA expression level in the model control group decreased significantly (*p* < 0.01), in comparison with that in the normal control group. This result demonstrated that the binding activity of insulin to IR is restricted. Wang et al. (2018) also reported that a high-energy diet decreased the mRNA expression of IR [32]. Owing to the reduced mRNA expression of IR, the IRS-2 (Figure 7c), PI3K (Figure 7d), and PKB (Figure 7e) mRNA expression levels in the model control group decreased significantly (*p* < 0.01). After the intervention with the SPED, the mRNA expression levels of IR, IRS-2, PI3K, and PKB increased significantly (*p* < 0.01), while GSK-3β (Figure 7f) mRNA expression level decreased significantly (*p* < 0.01). Additionally, GSK-3β is a negative regulatory protein, which regulates the activation of glycogen synthase [35]. The reduced mRNA expression of GSK-3β in SPED groups promoted the utilization of blood glucose and hepatic glycogen synthesis. Taken together, our study confirmed that SPED could promote hepatic glycogen synthesis by activating the PI3K/PKB/GSK-3β signaling pathway at the transcriptional level. 

## 3. Materials and Methods 

### 3.1. Materials

*E. prolifera* was harvested in February 2016 in Qingdao, China. Dialysis membrane (molecular weight cut-off of 1000 Da) was obtained from the Beijing Solarbio Science & Technology Co. (Beijing, China). Dextran standards (3.65, 5, 12, 25 kDa), were purchased from Sigma Co. (St. Louis, MO, USA). All other chemicals and reagents were of analytical grade. 

### 3.2. Preparation and Characterization of SPE

Degradase for sulfated polysaccharide was prepared as described by Cui et al. (2018) [36]. Briefly, *Alteromonas* sp. A321 was cultivated and its extracellular supernatant was extracted. The supernatant was brought to 60% (*w*/*v*) saturation with (NH_4_)_2_SO_4_. Then, the degradase precipitate was dissolved in 20 mM phosphate buffer saline (pH 7.0). The sulfated polysaccharide was extracted using hot water and hydrolyzed by the degradase (1.08 U/mL, enzyme-substrate ratio of 1:6) for 10 h under the conditions of 35 °C, pH 6.8–7.0. Then the enzymatic hydrolysate was quenched by heating at 100 °C for 10 min. After ethanol precipitation, the supernatant was concentrated, dialyzed, and lyophilized. The obtained powder was referred to as SPE.

The molecular weights (Mws) of the polysaccharides were determined by using an Agilent 1260 HPLC system (Wilmington, DE, USA) equipped with PL Aquagel-OH 30 column (0.75 × 30 cm) and a refractive index detector. The column was eluted with 0.2 M NaNO_3_ and 0.01 M NaH_2_PO_4_ at a flow rate of 0.6 mL/min. The molecular weights of all samples were estimated by referencing the calibration curve made from dextran standards [36]. The monosaccharide composition was determined by reversed-phase HPLC after pre-column derivatization. Moreover, the sulfate content was analyzed according to the method reported by Therho & Hartiala (1971) [37].

### 3.3. Synthesis and Characterization of the SPED

Briefly, 1 mL of 0.5 M CrCl_3_·6H_2_O was added dropwise under continuous stirring to 2% (*w*/*v*) SPE and 0.15% (*w*/*v*) sodium citrate aqueous solution. During the process, 1 M NaOH was used to control pH of the system around 6. The solution was heated at 60 °C for 3 h and centrifuged at 4800 r/min for 10 min. The supernatant was concentrated and then dialyzed in distilled water for 48 h to remove the unbound Cr^3+^. Finally, the dialysate was concentrated and lyophilized to obtain the SPED. 

The molecular weight, monosaccharide composition, and sulfate content of the SPED were determined by methods that were described in Section 3.2. The circular dichroism (CD) spectra of SPE and SPED solutions (0.5 mg/mL) were determined using the J-180 CD spectrometer (JASCO, Japan). Data were collected from 190 to 300 nm at 1-nm intervals. Two milligrams of samples were mixed with 200 mg KBr. The Fourier transform infrared spectroscopy (FTIR) data were recorded in the wavelength range of 4000–400 cm^−1^, using MAGNA-IR 560 E.S.P (Nicolet, USA).

### 3.4. Animals

Male ICR (20 ± 2 g) and C57BL/6J mice (18 ± 2 g), 4–5 weeks, were purchased from Vital River Laboratory Animal Center (Beijing, China; Licensed ID: SCXK2009-0007). All mice were housed under controlled conditions (50% humidity, 24 ± 1 °C, and 12 h light-dark cycle). The mice were allowed free access to food and water. The mice were acclimatized for five days to the new environment before experiment. All animal experiments were carried out in accordance with internationally valid guidelines and experimental protocols were prior approved by animal ethics committee as per the guidelines of the Standards for Laboratory Animals of China (GB 14922-94, GB 14923-94, and GB/T 14925-94).

### 3.5. Safety Evaluation of the SPED

In order to carry out acute toxicity experiment, forty male ICR mice (four-week-old) were randomly divided into normal control group, low dose group, medium dose group, and high dose group. The normal control group was intragastrically given 0.9% NaCl solution. The dose-groups were intragastrically given different concentrations of the SPED (0.07, 0.40, and 2.38 g/kg). All mice were monitored for 14 d and kept under regular observation for any mortality or behavioral changes. 

For the sub-acute toxicity experiment, 40 male ICR mice (four-week-old) were randomly divided into normal control group, low dose group, medium dose group, and high dose group. The normal control group was given 0.9% NaCl solution. The dose-groups were intragastrically given different concentration of the SPED (125, 250, and 500 mg/kg). All the mice were sacrificed after four weeks of administration of SPED. The organs were collected for histological analysis.

### 3.6. Induction of Type 2 Diabetic Mice and Experimental Design

Eighty male C57BL/6J mice were randomly assigned to normal control group, model control group, positive control group, negative control group, low SPE group, high SPE group, low SPED group, and high SPED group. The specific doses of the samples are shown in Table 2. The normal control group was maintained on a normal chow diet. Other groups were treated with a high-fat and high-sucrose diet. After 16 weeks of treatment, all the mice were sacrificed, and pancreas and livers were isolated for further analysis.

#### 3.6.1. Oral Glucose Tolerance Test

The blood glucose content was determined using a commercial kit (Biosino, Beijing, China). All mice were orally administered with glucose solution at a dose of 2 g/kg, after five weeks of treatment. An oral glucose tolerance test was conducted according to blood glucose levels at 0, 0.5, 1, and 2 h after glucose administration. The AUC defined as an oral glucose tolerance was calculated according to Formula (1).

AUC = 0.25 × A + 0.5 × B + 0.75 × C + 0.5 × D (A, B, C, and D represent blood glucose level at 0, 0.5, 1, and 2 h, respectively)(1)

#### 3.6.2. Evaluation of Serum Insulin Level

At the end of the experiment, blood samples were collected from the orbital sinus. The serum insulin level was assessed using an insulin ELISA kit (Invitrogen, Carlsbad, USA). HOMA-IR and QUICKI were calculated according to Formulas (2) and (3), respectively.

HOMIA − IR = fasting blood glucose × serum insulin/22.5(2)

QUICKI = 1/[lg (fasting blood glucose) + lg (serum insulin)](3)

#### 3.6.3. Microscopic Structure of Pancreas Islet 

The tails of the pancreas were fixed in 4% paraformaldehyde, paraffin embedded, sectioned, stained with hematoxylin and eosin (H&E). Microscopic structures of the pancreas islets were observed and photographed using an optical microscope (BH-2 Olympus, Japan).

#### 3.6.4. Evaluation of Hepatic Glycogen Content

The livers (80–100 mg) were homogenized with NaOH and heated for 30 min in a boiling water bath. After cooling in an ice bath, samples were diluted 96-fold in distilled water. The glycogen concentration was measured by the glycogen reagent kit, which was purchased from Jiancheng Bioengineering Institute (Nanjing, China). 

#### 3.6.5. Quantitative Real-Time PCR Analysis

The mRNA expression levels of insulin receptors (IR), insulin receptor substrate-2 (IRS-2), phosphatidylinositol 3 kinase (PI3K), protein kinase B (PKB), and glycogen synthase kinase 3β (GSK-3β) were examined by quantitative real-time PCR reaction. Total RNA from the liver was isolated using a commercial kit (OMEGA, USA). The purity of the RNA was assessed based on the ratio of the readings at 260 nm and 280 nm in a spectrophotometer. The cDNA was reverse-transcribed from total RNA, using a first-strand cDNA synthesis kit (Thermo Fisher Scientific, USA). Finally, cDNA samples were diluted 1:5 and real-time PCR was performed using iQ5 SYBR Green Supermix and a quantitative real-time PCR thermocycler (BioRad, USA). The primer sequences used for amplification were as follows: 

IR, (F) 5’-TGTCCCCAGAAAAACCTCTTCA-3’, (R) 5’-AAGGGATCTTCGCTTTCGGG-3’; IRS-2, (F) 5’-CACAATTCCAAGCGCCACAA-3’, (R) 5’-CATCACCTCCTCCCAGGGTA-3’; PI3K, (F) 5’-TCACTACAACCACCAGGAGC-3’, (R) 5’-CATCTGACTAACGCCACGAG-3’;

PKB, (F) 5’-AGGAAGGGGTGCCTGGTAT-3’, (R) 5’-GTCGCCAACAGTCTGAAGC-3’; GSK-3β, (F) 5’-ACCCTCATTACCTGACCTT-3’, (R) 5’-TCGGCAGACAATTCAACTC-3’; β-actin, (F) 5’-CAAGGCATTGCTGACAGGATG-3’, (R) 5’-TGCTGATCCACATCTGCTGG-3’.

### 3.7. Statistical Analysis

All data are expressed as a mean ± standard deviation. Statistical analyses were performed with SPSS version 12.0. Comparisons between the groups were performed by the one-way analysis of variance (ANOVA) and least significant difference (LSD) test. A difference of *p* < 0.05 was considered to indicate statistical significance.

## 4. Conclusions

In this study, a novel SPED (6.4 kDa) was first synthesized. It is a kind of high rhamnose-containing polysaccharide (58.21%), with 24.38% sulfate ester. Structural characteristics indicated that chromium (III) was chelated with the hydroxyl, carboxyl, and sulfate ester of SPE. Safety evaluation showed that SPED caused no damage to mice. Anti-diabetic experiment demonstrated that it could improve the glucose tolerance capacity, insulin sensitivity, and impaired pancreatic β-cells. Furthermore, quantitative real-time PCR confirmed that it could promote hepatic glycogen synthesis by activating the PI3K/PKB/GSK-3β signaling pathway at the transcriptional level. The SPED could be a novel rhamnan-type sulfated polysaccharide derivative to ameliorate metabolic syndrome in type 2 diabetes.

## Figures and Tables

**Table 1:** Chemical characteristics of the samples.

Samples	Total Sugar %	Sulfate (%)	Rhamnose (mol/%)	Glucose (mol/%)	Xylose (mol/%)	Glucuronic Acid (mol/%)	Mw (kDa)
SPE	70.1	25.9	59.8	3.1	17.9	19.1	4.8
SPED	71.6	24.4	58.2	4.3	16.9	20.5	6.4

**Table 2:** Grouping design and dosages of type 2 diabetic experiment.

Groups	Samples	Dose (mg/kg)	Equivalent to Cr^3+^ (µg/kg)
Normal Control	NaCl (0.9%)	--	
Model Control	NaCl (0.9%)	--	
Positive Control	Picolinic	4.5	560
Negative Control	CrCl_3_·6H_2_O	2.9	560
Low SPE	SPE	5	
High SPE	SPE	20	
Low	SPED	5	140
High	SPED	20	560

## References

[1] Arrese M. (2010). Nonalcoholic fatty liver disease: Liver disease: An overlooked complication of diabetes mellitus. Nat. Rev. Endocrinol..

[2] Shaw J.E., Sicree R.A., Zimmet P.Z. (2010). Global estimates of the prevalence of diabetes for 2010 and 2030. Diabetes Res. Clin. Pract..

[3] Ibrahim M.A., Habila J.D., Koorbanally N.A., Islam M.S. (2016). Butanol fraction of *Parkia biglobosa* (Jacq.) G. Don leaves enhance pancreatic β-cell functions, stimulates insulin secretion and ameliorates other type 2 diabetes-associated complications in rats. J. Ethnopharmacol..

[4] Lewicki S., Zdanowski R., Krzyzowska M., Lewicka A., Debski B., Niemcewicz M., Goniewicz M. (2014). The role of chromium III in the organism and its possible use in diabetes and obesity treatment. Ann. Agric. Environ. Med..

[5] Peng M., Yang X. (2015). Controlling diabetes by chromium complexes: The role of the ligands. J. Inorg. Biochem..

[6] Tan G.Y., Zheng S.S., Zhang M.H., Feng J.H., Xie P., Bi J.M. (2008). Study of oxidative damage in growing-finishing pigs with continuous excess dietary chromium picolinate intake. Biol. Trace Elem. Res..

[7] Ren R., Gong J., Zhao Y., Zhuang X., Ye Y., Huang F., Lin W. (2018). Sulfated polysaccharide from *Enteromorpha prolifera* suppresses SREBP-1c and ACC expression to lower serum triglycerides in high-fat diet-induced hyperlipidaemic rats. J. Funct. Foods.

[8] Qi X., Mao W., Gao Y., Chen Y., Chen Y., Zhao C., Li N., Wang C., Yan M., Lin C. (2012). Chemical characteristic of an anticoagulant-active sulfated polysaccharide from *Enteromorpha clathrate*. Carbohydr. Polym..

[9] Cui J., Li Y., Yu P., Zhan Q., Wang J., Chi Y., Wang P. (2018). A novel low molecular weight *Enteromorpha* polysaccharide-iron (III) complex and its effect on rats with iron deficiency anemia (IDA). Int. J. Biol. Macromol..

[10] Zhang Y., Zhang J., Mo X., Lu X., Zhang Y., Qin L. (2010). Modifcation, characterization and structure-anticoagulant activity relationships of persimmon polysaccharides. Carbohydr. Polym..

[11] Combo A.M.M., Aguedo M., Quiévy N., Danthine S., Goffin D., Jacquet N., Blecker C., Devaux J., Paquot M. (2013). Characterization of sugar beet pectic-derived oligosaccharides obtained by enzymatic hydrolysis. Int. J. Biol. Macromol..

[12] Shi J., Cheng C., Zhao H., Jing J., Gong N., Lu W. (2013). In vivo anti-radiation activities of the *Ulva pertusa* polysaccharides and polysaccharide-iron (III) complex. Int. J. Biol. Macromol..

[13] Wang C., Chen Z., Pan Y., Gao X., Chen H. (2017). Anti-diabetic effects of *Inonotus obliquus* polysaccharides-chromium (III) complex in type 2 diabetic mice and its sub-acute toxicity evaluation in normal mice. Food Chem. Toxicol..

[14] Ranjbar B., Gill P. (2009). Circular dichroism techniques: Biomolecular and nano-structural analyses—A review. Chem. Biol. Drug Des..

[15] Wang J., Chen H., Wang Y., Xing L. (2015). Synthesis and characterization of a new *Inonotus obliquus* polysaccharide-iron (III) complex. Int. J. Biol. Macromol..

[16] Park J.W., Chakrabarti B. (1978). Optical characteristics of carboxyl group in relation to the circular dichroic properties and dissociation constants of glycosamino-glycans. Biochim. Biophys. Acta BBA-Mol. Basis Dis..

[17] Zhao X., Yu G., Guan H., Yue N., Zhang Z., Li H. (2007). Preparation of low-molecular-weight polyguluronate sulfate and its anticoagulant and anti-inflammatory activities. Carbohydr. Polym..

[18] Mao W.J., Fang F., Li H.Y., Qi X.H., Sun H.H., Chen Y., Guo S.D. (2008). Heparinoid-active two sulfated polysaccharides isolated from marine green alage *Monostroma nitidum*. Carbohydr. Polym..

[19] Yu Y., Li Y., Du C., Mou H., Wang P. (2017). Compositional and structural characteristics of sulfated polysaccharide from *Enteromorpha prolifera*. Carbohydr. Polym..

[20] Saheed S., Oladipipo A.E., Abdulazeez A.A., Olarewaju S.A., Ismaila N.O., Emmanuel I.A., Fatimaha Q.D., Aisha A.Y. (2015). Toxicological evaluations of *Stigma maydis* (corn silk) aqueous extract on hematological and lipid parameters in Wistar rats. Toxicol. Rep..

[21] Wu X.Y., Li F., Xu W.D., Zhao J.L., Zhao T., Liang L.H., Yang L.Q. (2011). Anti-hyperglycemic activity of chromium (III) malate complex in alloxan-induced diabetic rats. Biol. Trace Elem. Res..

[22] Li F., Wu X., Zhao T., Zhang M., Zhao J., Mao G., Yang L. (2011). Anti-diabetic properties of chromium citrate complex in alloxan-induced diabetic rats. J. Trace Elem. Med. Biol..

[23] Stallings D.M., Hepburn D.D.D., Hannah M., Vincent J.B., O’Donnell J. (2006). Nutritional supplement chromium picolinate generates chromosomal aberrations and impedes progeny development in Drosophila melanogaster. Mutat. Res..

[24] Hepburn D.D.D., Vincent J.B. (2003). Tissue and subcellular distribution of chromium picolinate with time after entering the bloodstream. J. Inorg. Biochem..

[25] Mazzola N. (2012). Review of current and emerging therapies in type 2 diabetes mellitus. Am. J. Manag. Care.

[26] Khan H.B., Vinayaqam K.S., Palanivelu S., Panchanadham S. (2012). Ameliorating effect of *Semecarpus anacardium* Linn. nut milk extract on altered glucose metabolism in high fat diet STZ induced type 2 diabetic rats. Asian Pac. J. Trop. Med..

[27] Dabhi B., Mistry K.N. (2015). Oxidative stress and its association with TNF-α-308 G/C and IL-1α-889C/T gene polymorphisms in patients with diabetes and diabetic nephropathy. Gene.

[28] El-Azab M.F., Attia F.M., El-Mowafy A.M. (2011). Novel role of curcumin combined with bone marrow transplantation in reversing experimental diabetes: Effects on pancreatic islet regeneration, oxidative stress, and inflammatory cytokines. Eur. J. Pharmacol..

[29] Zhang C., Chen H., Bai W. (2018). Characterization of *Momordica charantia* L. polysaccharide and its protective effect on pancreatic cells injury in STZ-induced Diabetic mice. Int. J. Biol. Macromol..

[30] Hu S., Chang Y., Wang J., Xue C., Li Z., Wang Y. (2013). Fucosylated chondroitin sulfate from sea cucumber in combination with rosiglitazone improved glucose metabolism in the liver of the insulin-resistant mice. Biosci. Biotechnol. Biochem..

[31] Jung U.J., Baek N.I., Chung H.G., Bang M.H., Yoo J.S., Jeong T.S., Lee K.T., Kang Y.J., Lee M.K., Kim H.J. (2007). The anti-diabetic effects of ethanol extract from two variants of *Artemisia princeps* Pampanini in C57BL/KsJ-db/db mice. Food Chem. Toxicol..

[32] Wang K., Wang H., Liu Y., Shui W., Wang J., Cao P., Wang H., You R., Zhang Y. (2018). *Dendrobium officinale* polysaccharide attenuates type 2 diabetes mellitus via the regulation of PI3K/Akt-mediated glycogen synthesis and glucose metabolism. Funct. Foods.

[33] Saltiel A.R., Kahn C.R. (2001). Insulin signaling and the regulation of glucose and lipid metabolism. Nature.

[34] Chang L., Chiang S.H., Saltiel A.R. (2004). Insulin signaling and the regulation of glucose transport. Mol. Med..

[35] Coops K.D., White M.F. (2012). Regulation of insulin sensitivity by serine/threonine phosphorylation of insulin receptor substrate proteins IRS1 and IRS2. Diabetologia.

[36] Cui J., Li Y., Wang S., Chi Y., Hwang H., Wang P. (2018). Directional preparation of anticoagulant-active sulfated polysaccharides from *Enteromorpha prolifera* using artificial neural networks. Sci. Rep..

[37] Terho T.T., Hartiala K. (1971). Method for determination of the sulfate content of glycosaminoglycans. Anal. Biochem..

